# From Cyanosis to Extensive Systemic‐to‐Pulmonary Collaterals Formation: A Case With an Iatrogenic Connection of the Inferior Vena Cava to the Left Atrium

**DOI:** 10.1002/ccr3.71169

**Published:** 2025-10-07

**Authors:** Akram Nakhaee, Roya Sattarzadeh Badkoubeh, Mehrzad Rahmanian, Maryam Roozitalab

**Affiliations:** ^1^ Cardiology Department, School of Medicine Imam Khomeini Hospital Complex, Tehran University of Medical Sciences Tehran Iran; ^2^ Department of Cardiovascular Surgery Imam Khomeini Hospital Complex, Tehran University of Medical Sciences Tehran Iran

**Keywords:** atrial septal defects (ASD), cyanosis, inferior vena cava (IVC), left atrium (LA)

## Abstract

Atrial septal defect (ASD) is a prevalent congenital heart abnormality that can be effectively repaired through either surgical or interventional procedures. We decide to describe a 50‐year‐old male with a history of dyspnea, cyanosis, and hypoxemia. A remarkable past medical history is that he underwent surgical correction of an ASD at the age of 6. Because of an initial misdiagnosis of Eisenmenger syndrome, he recently received palliative therapy, including therapeutic phlebotomy. Subsequent coronary computed tomography angiography (CTA) revealed systemic‐to‐pulmonary collaterals (SPCs) and an iatrogenic diversion of the inferior vena cava (IVC) to the left atrium (LA). A contrast study in transesophageal echocardiography confirmed the IVC's aberrant pathway to the LA. The diagnostic pathway and rationale behind these findings underscore the significance of multimodality imaging and reevaluation of the initial diagnostic impression.

**Summary**

- Iatrogenic deviation of the inferior vena cava (IVC) to the left atrium (LA) is a very rare complication.
- We presented a man with a history of ASD closure in childhood, progressive cyanosis, and clubbing.
- The use of multimodality imaging revealed a diversion of the IVC into the LA.

## 1 Introduction

Atrial septal defect (ASD) represents a prevalent congenital cardiac anomaly that can be effectively managed through either surgical or non‐surgical interventional approaches. The most prevalent complications following ASD repair encompass atrial arrhythmias, mediastinal bleeding, and stroke.

Iatrogenic connection of the inferior vena cava (IVC) to the left atrium (LA) is an uncommon and exceedingly rare complication. This complication has been documented following the closure of sinus venous atrial septal defects (ASDs), with one contributing factor being the misidentification of large eustachian valves as part of the margin of the ASD [1]. Early postoperative diagnosis of IVC‐to‐LA communication is possible. Still, it may remain undetected for an extended period, presenting later with features like clubbing associated with paradoxical embolization and cyanosis [2]. Cyanosis results from a right‐to‐left shunt when systemic venous return bypasses the pulmonary circulation, leading to increased levels of deoxygenated hemoglobin in arterial blood, which may affect multiple organ systems [3]. Subsequent cyanosis, coupled with reduced pulmonary blood flow and increased oxygen demand, may contribute to the development of collateral vessels [4]. This report presents an extremely interesting case illustrating the iatrogenic IVC‐to‐LA connection, highlighting its association with cyanosis and extensive mediastinal collateral formation.

## 2 Case History

**FIGURE 1:**
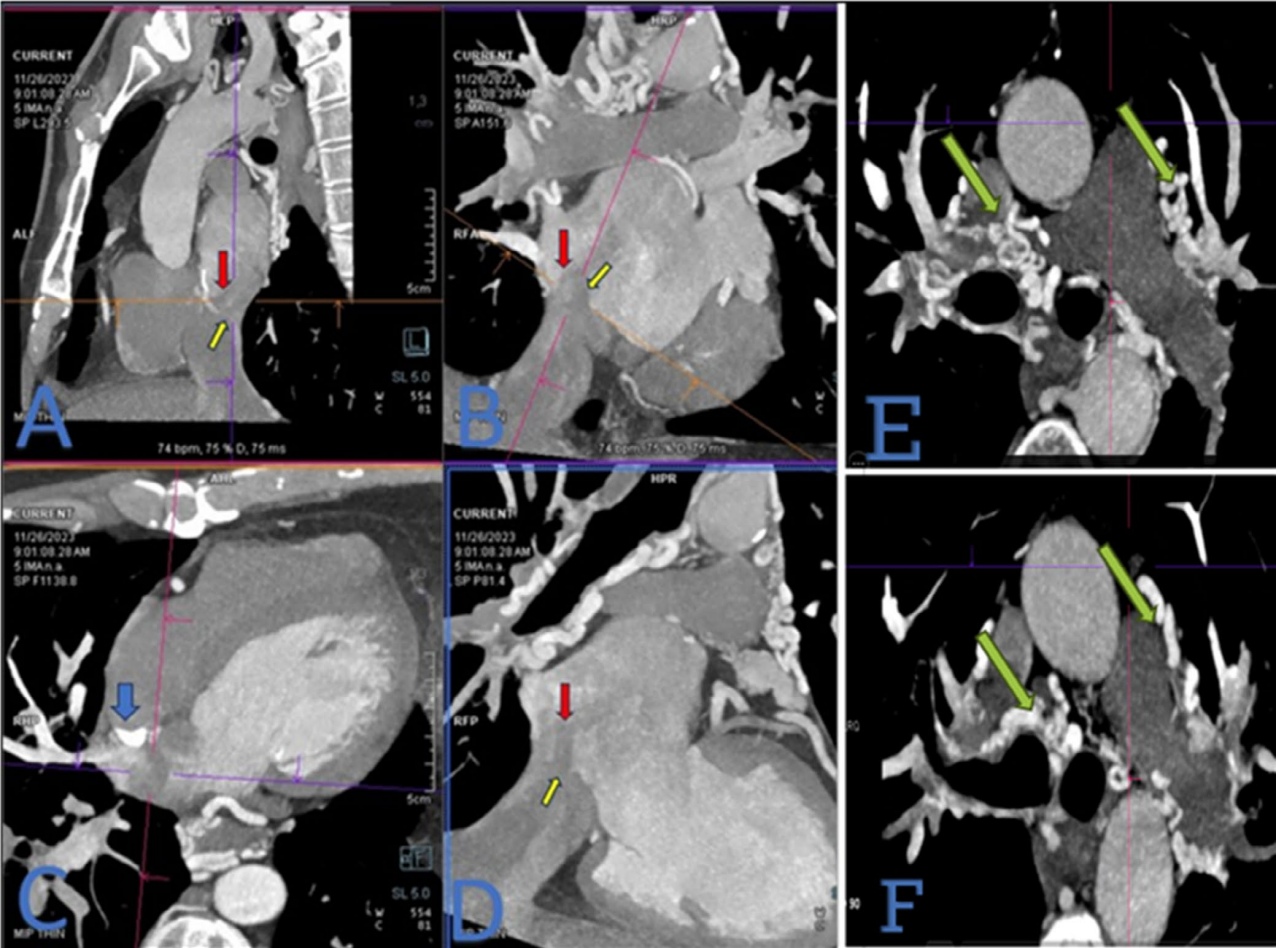
(A) Oblique sagittal reformatted image. (B) Coronal oblique reformatted image at the inferior vena cava (IVC) entrance level. (C) Axial oblique reformatted image. (D) Oblique reformatted image illustration. (E) and (F) Axial images at the level of the main pulmonary artery. A calcified surgical patch (blue arrow) is seen between the atria to close a secundum‐type atrial septal defect (ASD). However, the IVC diverts to the left atrium (LA) (red arrow) due to iatrogenic misalignment of the surgical patch via a remnant ASD (yellow arrow). Systemic‐to‐pulmonary collaterals are noted without any stenosis in the pulmonary arteries (green arrow).

**FIGURE 2:**
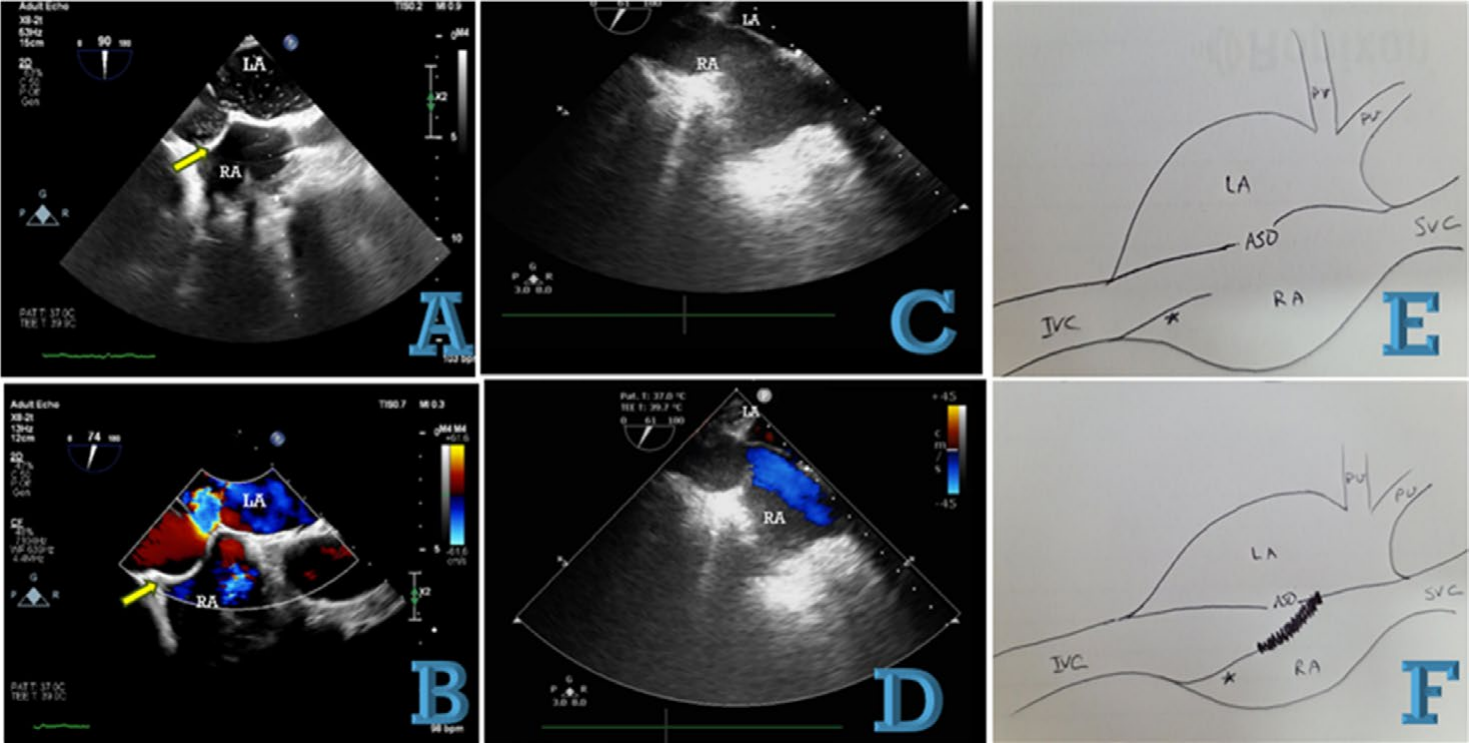
(A) Transesophageal echocardiography at 90 degrees and biplane view after contrast injection from the leg. All bubbles pass to the left atrium (LA). (B) Color Doppler study at 74 degrees reveals a right‐to‐left shunt on the right side of the previous patch (yellow arrow) via a remnant atrial septal defect (ASD). (C) Post‐surgery image showing the patch being cut and the ASD repaired. (D) Color Doppler study demonstrating normal color flow in the right atrium (RA) via the inferior vena cava (IVC). (E) Schematic view of the probable pre‐first surgery scenario. (F) Schematic view of the post‐first surgery situation.

The patient presented with dyspnea and cyanosis in both the upper and lower extremities, accompanied by hypoxemia. The patient's medical history includes surgical repair of an atrial septal defect (ASD) in childhood and prolonged therapeutic phlebotomy, potentially due to a misdiagnosis of Eisenmenger syndrome after the manifestation of the described symptoms. Due to worsening dyspnea and the onset of atypical chest pain, the patient underwent a diagnostic evaluation, including coronary computed tomography angiography (CCTA). The coronary computed tomography angiography (CCTA) identified significant systemic‐to‐pulmonary collaterals originating from the aorta and its branches, predominantly the left and right subclavian arteries, the left circumflex artery, and the descending aorta to pulmonary arteries (Figure 1). Remarkably, in the extracoronary evaluation, the inferior vena cava (IVC) was observed to divert to the left atrium (LA) just posterior to the surgical patch. As expected, systemic‐to‐pulmonary collaterals were noted without any stenosis in the pulmonary arteries (Video 1). As anticipated, systemic‐to‐pulmonary collaterals are consistently observed in the context of pulmonary atresia or diminished pulmonary blood flow. Contrast‐enhanced TEE was performed to evaluate the interatrial septum, inferior vena cava (IVC), and other structures better. The Eustachian valve was observed to be attached to the interatrial septum, proximal to the atrial septal defect (ASD), potentially directing IVC flow into the left atrium (LA) via the unrepaired ASD. As part of our diagnostic plan, agitated saline bubbles were injected through the lower limb. The bubbles traveled entirely from the IVC to the LA, while the right atrium (RA) remained unoccupied (Figure 2). Following a multidisciplinary heart team discussion, including cardiac surgeons, surgical intervention was scheduled to address the pathology. Intraoperatively, the surgeon identified that the elongated Eustachian valve was sutured to the septum via a dense surgical patch. The surgeon redirected the inferior vena cava (IVC) to the right atrium (RA) by cutting the previous patch and repairing the atrial septal defect (ASD) (Video 2). Additionally, the fistulous termination of the left circumflex (LCx) artery into the pulmonary artery was ligated.

**VIDEO 1:** Cardiac CT angiography with multiplanar reconstruction (axial, coronal, and sagittal planes) and volume‐rendered reconstruction demonstrates multiple systemic‐to‐pulmonary collaterals and an iatrogenic diversion of the inferior vena cava (IVC) to the left atrium (LA). Video content can be viewed at https://onlinelibrary.wiley.com/doi/10.1002/ccr3.71169.

**VIDEO 2:** Transesophageal echocardiography reveals dilation of the distal portion of the inferior vena cava (IVC) to the left atrium (LA). Color Doppler study confirms the presence of a remnant atrial septal defect (ASD) with a continuous right‐to‐left shunt. Contrast study shows bubble passages from the IVC to the LA, indicating the shunt's patency. Video content can be viewed at https://onlinelibrary.wiley.com/doi/10.1002/ccr3.71169.

## 3 Conclusion and Results

This case underscores how multimodality imaging can be highly valuable in clarifying complex anatomy and missed diagnoses, particularly when traditional echocardiographic windows are suboptimal. Although not universally necessary, it can play an essential role in select cases of diagnostic uncertainty. The incidental finding of an iatrogenic diversion of the inferior vena cava (IVC) to the left atrium (LA), coupled with extensive systemic‐to‐pulmonary collaterals, underscores the necessity for a thorough reevaluation of initial diagnostic impressions, especially in patients with a history of congenital heart disease and new or persistent symptoms such as cyanosis and hypoxemia. Early and accurate identification of such anomalies can significantly impact patient management and outcomes, emphasizing the value of advanced imaging techniques in contemporary cardiology.

## 4 Discussion

Iatrogenic diversion of the inferior vena cava (IVC) to the left atrium (LA) can be diagnosed intraoperatively or after a prolonged diagnostic interval extending into adulthood [1]. The time to detect inadvertent deviation of the IVC to the LA varies among reports. Thompson et al. [5] and Bjork et al. [6] reported detection during recovery and immediately post‐surgery, while Desai et al. [7] and Jain et al. [8] reported detection intervals ranging from 2 to 30 years post‐operation.

The primary common risk factors for technical error of iatrogenic diversion of the IVC to LA include a low‐lying ASD, a posteroinferior position of the ASD, sinus venosus type ASD, and an elongated Eustachian valve [2].

Analogous to numerous cardiac conditions, echocardiography is frequently employed as the primary diagnostic tool to detect iatrogenic deviation of the inferior vena cava (IVC) to the left atrium (LA) [1].

In contrast to prior literature, in our study, we incidentally observed iatrogenic deviation of the inferior vena cava (IVC) into the left atrium (LA) using cardiac computed tomography angiography (CCTA). Our case stands out due to the unique size and shape of the collateral vessels associated with prominent systemic‐to‐pulmonary collaterals. The etiology of prominent systemic‐to‐pulmonary collaterals can be categorized into congenital and acquired forms. Congenital collaterals often occur in the context of cyanotic congenital diseases such as tetralogy of Fallot and pulmonary atresia. Acquired collaterals, on the other hand, develop in settings of decreased pulmonary flow or increased pulmonary vascular resistance, such as chronic thromboembolic pulmonary hypertension (CTEPH) [9, 10].

## Author Contributions

**Akram Nakhaee:** writing – original draft, writing – review and editing. **Roya Sattarzadeh Badkoubeh:** writing – original draft, writing – review and editing. **Mehrzad Rahmanian:** writing – original draft, writing – review and editing. **Maryam Roozitalab:** data curation, supervision, writing – original draft, writing – review and editing.

## Ethics Statement

The authors have nothing to report.

## Consent

Written consent was obtained from the patient for the publication of the case report as well as the relevant clinical information, examination images, and data.

## Conflicts of Interest

The authors declare no conflicts of interest.

## Data Availability

No datasets were generated or analyzed during the current study.
